# Adipose-derived mesenchymal stem cells promote the survival of fat grafts via crosstalk between the Nrf2 and TLR4 pathways

**DOI:** 10.1038/cddis.2016.261

**Published:** 2016-09-08

**Authors:** Xiaosong Chen, Liu Yan, Zhihui Guo, Zhaohong Chen, Ying Chen, Ming Li, Chushan Huang, Xiaoping Zhang, Liangwan Chen

**Affiliations:** 1 Department of Plastic Surgery, The Union Hospital of Fujian Medical University, 29 Xinquan Road, Fuzhou, Fujian 350001, China; 2 Department of Stem Cell Research Institute, Fujian Medical University, Fuzhou, Fujian 350000, China; 3 Department of Burns Surgery, The Union Hospital of Fujian Medical University, 29 Xinquan Road, Fuzhou, Fujian 350001, China; 4 Institution of Interventional and Vascular surgery, Tongji Univerity, No 301 Middle Yan Chang Road, Shanghai 200072, China; 5 Department of Cardiac Surgery, The Union Hospital of Fujian Medical University, 29 Xinquan Road, Fuzhou, Fujian 350001, China

## Abstract

Autologous fat grafting is an effective reconstructive surgery technique; however, its success is limited by inconsistent graft retention and an environment characterized by high oxidative stress and inflammation. Adipose-derived stem cells (ADSCs) increase the survival of fat grafts, although the underlying mechanisms remain unclear. Here, TLR4^−/−^ and Nrf2^−/−^ mice were used to explore the effects of oxidative stress and inflammation on the viability and function of ADSCs *in vitro* and *in vivo*. Enrichment of fat grafts with ADSCs inhibited inflammatory cytokine production, enhanced growth factor levels, increased fat graft survival, downregulated NADPH oxidase (NOX)1 and 4 expression, increased vascularization and reduced ROS production in a manner dependent on toll-like receptor (TLR)-4 and nuclear factor erythroid 2-related factor 2 (Nrf2) expression. Immunohistochemical analysis showed that exposure to hypoxia enhanced ADSC growth and promoted the differentiation of ADSCs into vascular endothelial cells. Hypoxia-induced inflammatory cytokine, growth factor and NOX1/4 upregulation, as well as increased ROS production and apoptosis in ADSCs were dependent on TLR4 and Nrf2, which also modulated the effect of ADSCs on promoting endothelial progenitor cell migration and angiogenesis. Western blot analyses showed that the effects of hypoxia on ADSCs were regulated by crosstalk between Nrf2 antioxidant responses and NF-*κ*B- and TLR4-mediated inflammatory responses. Taken together, our results indicate that ADSCs can increase the survival of fat transplants through the modulation of inflammatory and oxidative responses via Nrf2 and TLR4, suggesting potential strategies to improve the use of ADSCs for cell therapy.

Autologous fat grafting, which is the transfer of subcutaneous fat from a donor site to a recipient site, is a commonly used procedure in both cosmetic and reconstructive surgery. However, the success of fat grafting is limited by the unpredictability of long-term graft retention, with a reported resorption rate of 10–90%.^1^ After enzymatic processing, harvested fat contains a mix of adipose-derived stem cells (ADSCs), vascular progenitor cells, endothelial cells and pericytes.^2^ The enrichment of grafted tissue with ADSCs, which are multipotent cells that can be induced to differentiate into many lineages, including adipogenic, chondrogenic, myogenic and osteogenic cells, is one of the most efficient techniques to improve the survival rate of fat grafts.^3, 4^ In addition, appropriate revascularization is an important factor determining graft survival, and new angiogenesis from the host vascular network is responsible for graft revascularization.^5, 6^ Engrafted ADSCs are also susceptible to cell death in an environment characterized by inflammation and oxidative stress.

Oxidative stress affects ADSCs and their regenerative capacity, and dysregulation of reactive oxygen species (ROS) production impairs ADSC expansion *in vitro* and the viability of engrafted ADSCs.^7^ However, low levels of ROS were shown to activate cellular processes involved in the normal physiological function of stem cells.^8^ Intracellular ROS are generated by the action of nicotinamide adenine dinucleotide phosphate (NADPH) oxidase enzymes (NOX) and passively by mitochondria.^9, 10^ Culture of ADSCs under hypoxic conditions increases their proliferative and migratory capacities and enhances the secretion of growth factors.^11, 12^ There are four NOX enzymes, of which NOX4 is predominantly expressed in ADSCs and modulates ROS signaling as well as the proliferation and differentiation of ADSCs.^13^

Toll-like receptors (TLRs) are pattern recognition receptors that respond to infection by recognizing pathogen-associated molecular patterns, triggering immune responses against invading micro-organisms.^14^ Twelve members of the TLR family have been identified in mammals, of which TLR4 is expressed on the cell surface. Nuclear factor erythroid 2-related factor 2 (Nrf2) is a transcriptional factor involved in cellular defenses against oxidative stress. Under normal conditions, Nrf2 localizes to the cytoplasm and binds to Kelch-like ECH-associated protein 1 (Keap-1), which mediates its proteasomal degradation, whereas Nrf2 activation induces its translocation to the nucleus to promote the transcription of target genes.^15^ Nrf2 activation promotes cell survival and protects against oxidative stress-induced damage, whereas disruption of Nrf2 signaling impairs the angiogenic capacity of endothelial cells and antioxidant gene expression, and enhances oxidative stress-mediated inflammation.^15^ Nrf2 regulates the expression of many antioxidant genes, including heme oxygenase-1 (HO-1), an antioxidant enzyme, through consensus *cis*-elements termed antioxidant-response elements.^16^

In the present study, TLR4^−/−^ and Nrf2^−/−^ mice were used to explore the effects of oxidative stress and inflammation on the viability and function of ADSCs and their role in improving the survival of fat grafts *in vitro* and *in vivo*.

## Results

### Effect of TLR4 and Nrf2 on inflammatory cytokine and growth factor secretion in ADSCs

**Figure 1:**
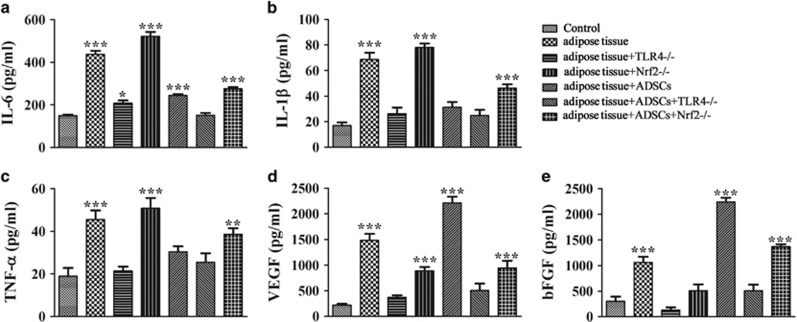
Effect of ADSC agonists on inflammatory cytokine and growth factor secretion in mice undergoing fat transplants. (**a**–**e**) The levels of TNF-*α*, IL-1*β* and IL-6, VEGF, and bFGF were measured by ELISA in the serum of wild-type (WT), TLR4^−/−^ and Nrf2^−/−^ mice with or without fat grafts and with or without the addition of ADSCs 2 weeks after transplantation (*n*=5). *P<0.05, **P<0.01, ***P<0.001 versus control; (**d** and **e**) Concentration of VEGF and bFGF in serum was also detected by ELISA (*n*=5). ***P<0.001 *versus* control group. Control, WT mice without fat graft

The effect of ADSC agonists on the secretion of inflammatory cytokines and growth factors was examined by measuring the levels of IL-6, TNF-*α*, IL-1*β*, VEGF and bFGF in the serum of mice with or without fat grafts 2 weeks after transplantation by ELISA. The results showed that addition of ADSCs attenuated inflammatory cytokine production and enhanced VEGF and bFGF production in mice receiving fat grafts (Figures 1a–e). The ADSC-induced decrease in inflammatory cytokines was enhanced by TLR4 deletion and partially restored by Nrf2 knockout (Figures 1a–c), whereas the ADSC-induced upregulation of growth factors was inhibited in TLR4^−/−^ mice and partly restored in Nrf2^−/−^ mice. Taken together, these results indicated that ADSCs modulate the changes in inflammatory cytokines and growth factors in fat grafts via a mechanism involving TLR4 and Nrf2.

### Effect of Nrf2 or TLR4 on ADSC-mediated survival of fat grafts

**Figure 2:**
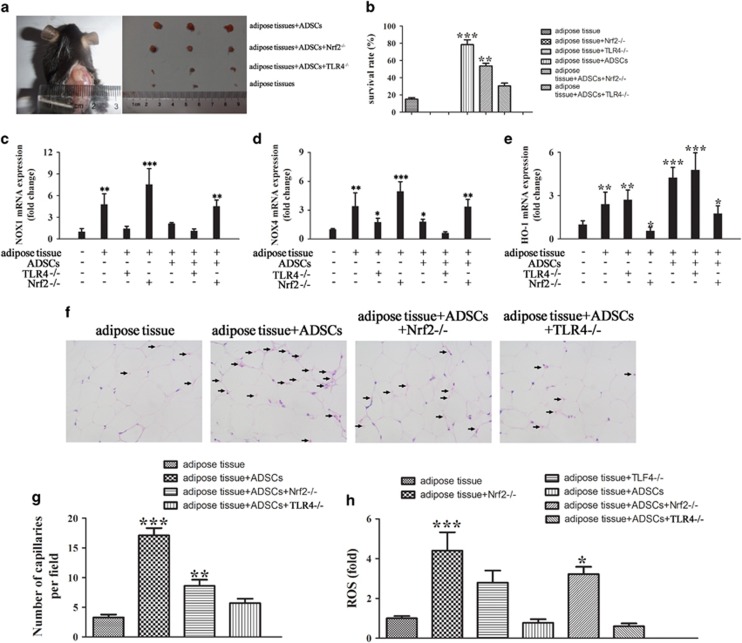
Effect of Nrf2 or TLR4 on ADSC-mediated survival of fat grafts. Nrf2^−/−^ or TLR4^−/−^ mice were injected subcutaneously in the left flank with fat tissues with or without 0.2 ml of 1 × 10^7^/ml GFP-labeled ADSCs. (**a**) Fat tissues were excised and representative images are shown. (**b**) The survival ratio of transplanted fat tissues was calculated using the following formula: survival volume/previous volume (0.5 ml). **P<0.01, ***P<0.001 *versus* control. (**c**–**e**) The expression of NOX1, NOX4 and HO-1 in transplanted adipose tissue 2 weeks after fat graft was measured by real-time PCR. *P<0.05, **P<0.01, ***P<0.001 *versus* control. (**f**) Hematoxylin and eosin (HE) staining of fat grafts (magnification, × 400) after 2 months. The arrowheads show the changes in the number of capillaries in the different groups. (**g**) The number of capillaries was counted from 10 fields per section taken from a central tissue section. **P<0.01, ***P<0.001 *versus* the adipose tissue group (adipose tissue group: fat graft without added ADSCs). (**h**) Measurement of ROS in adipose tissues 2 weeks after fat transplantation (*n*=5). *P<0.05, ***P<0.001 *versus* adipose tissue group

The survival of fat grafts was assessed in control and TLR4 or Nrf2 knockout mice receiving adipose tissues enriched with ADSCs. The results showed that ADSCs promoted the survival of fat grafts, whereas this effect was significantly decreased in Nrf2 and TLR4 knockout mice, in which grafts did not survive after 2 months in the absence of ADSCs (Figures 2a and b). Analysis of NOX1, NOX4 and HO-1 expression in transplanted adipose tissues by real-time PCR 2 weeks after transplantation showed that ADSCs downregulated NOX1 and NOX4 in mice receiving fat grafts, and this effect was enhanced by TLR4 knockout, whereas it was suppressed by Nrf2 knockout, which restored NOX1/4 levels to those observed in mice receiving untreated fat grafts (Figures 2c and d). ADSCs significantly upregulated HO-1, and this effect was enhanced by TLR4 deletion, whereas it was suppressed by Nrf2 deletion (Figure 2e). Hematoxylin and eosin staining of fat grafts 2 weeks after transplantation showed that addition of ADSCs increased the capillarization of fat tissues, whereas the increase in capillary density was decreased by TLR4 or Nrf2 knockout (Figure 2f). Quantification of the number of capillaries showed that ADSCs caused an approximately fourfold increase in the number of capillaries in adipose tissues, and this effect was partially suppressed in TLR4 and Nrf2 knockout mice (Figure 2g). In addition, Nrf2 and to a lesser extent TLR4 knockout increased ROS generation in adipose tissues 2 weeks after transplantation, whereas ADSC treatment partially suppressed this effect, restoring ROS levels (Figure 2g).

### Differentiation of ADSCs into vascular endothelial cells

**Figure 3:**
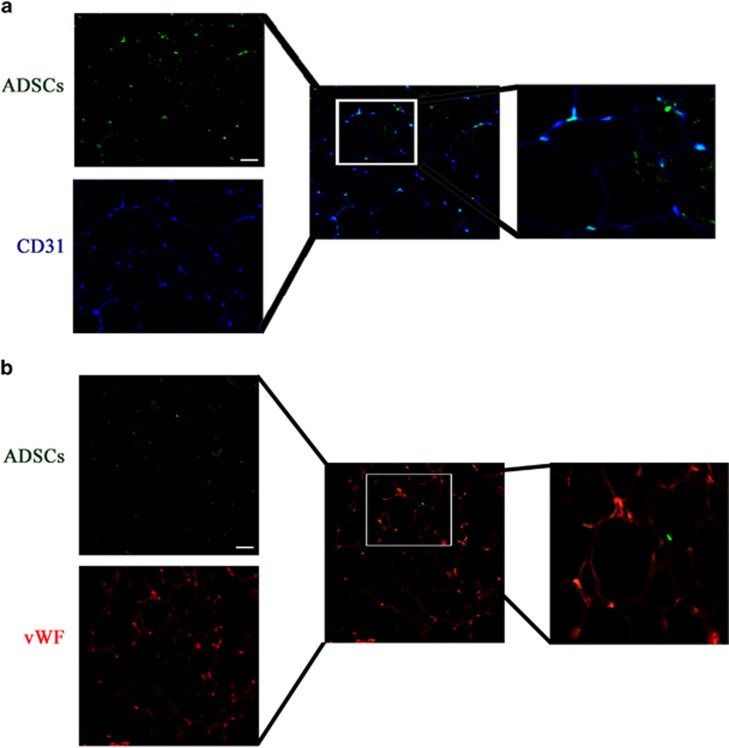
Origin of endothelial cells in surviving transplanted fat tissue. GFP-labeled ADSCs were detected by immunohistochemistry in sections from fat transplants. Representative images are shown. Transplant sections were stained with a Cy3-labeled mAb targeted against the endothelial cell marker CD31 (blue fluorescence) or phycoerythrin (red color) targeted against the endothelial cell marker vWF. The merged images show the co-localization of CD31 or vWF with GFP, indicating the differentiation of endothelial cells from GFP-labeled ADSCs (magnification, × 200). The result show that the survive ADSCs can differentiation into vascular endothelial cells in both **a** and **b** with different endothelial cell marker staining. Scale bar: 50 *μ*m

To determine the origin of endothelial cells (isolation and identification of endothelial progenitor cells (EPCs) were seen in Supplementary Figure S8) in surviving transplanted fat tissue, transplant sections were stained for the endothelial cell marker CD31 and analyzed by immunofluorescence. GFP-labeled ADSCs (Supplementary Figure S9) were detected in capillary-like structures and surrounding mature adipose tissue, as were CD31 and vWF stained neovascular capillary endothelial cells (stained in blue; Figure 3). Merging of images indicated the endothelial cells that had differentiated from GFP-labeled ADSCs, whereas the lack of co-localization of green and blue fluorescence indicated the potential differentiation of endothelial cells from EPCs.

### Effect of hypoxia on cell growth and differentiation

**Figure 4:**
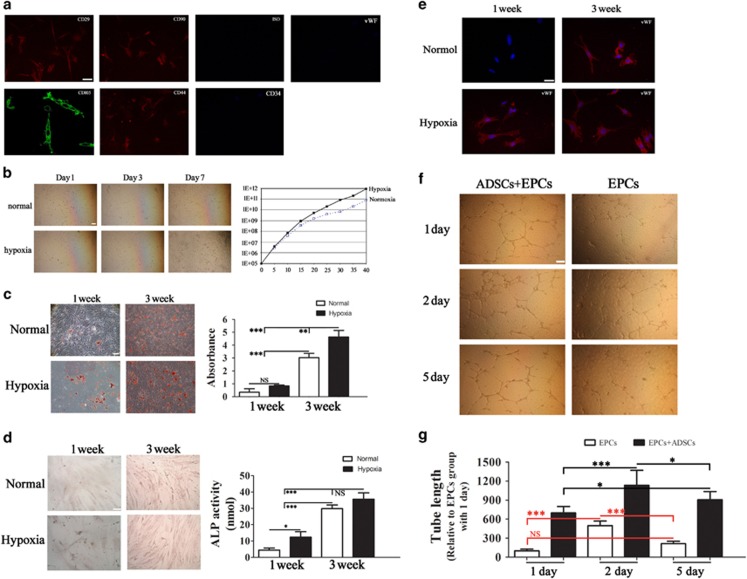
Characteristics of ADSCs and the effect of low oxygen tension on multipotent differentiation potential. (**a**) Detection of cell surface markers by immunofluorescence staining with antibodies against CD29, CD90, CD44, CD105, CD34 and vWF labeled with FITC (green color) or phycoerythrin (PE) (red color). A negative FITC and PE labeled mouse IgG isotype control was included (magnification: × 200). Scale bar: 5 *μ*m. (**b**) Phase contrast images of ADSCs showing morphological changes relative to the duration of the culture, and graph showing the growth of ADSCs under hypoxic and normoxic conditions over a period of 40 days (magnification, × 200). Scale bar: 20 *μ*m. (**c** and **d**) The differentiation potential of ADSCs was assessed by Oil-red-O (**c**) and alkaline phosphatase (ALP) (**d**) staining at 1 and 3 weeks after culture in adipogenic or osteogenic induction media, respectively, under hypoxic or normoxic conditions (magnification, × 400). Scale bar: 20 *μ*m. Quantitative analysis of fat drops extracted with isopropanol was performed with the Lipid Assay Kit and measurement of specific absorbance. Values represent the mean (S.E.) of three experiments. ALP activity was measured with an ALP activity assay. (**e**) The endothelial differentiation potential of ADSCs was analyzed by immunofluorescence detection of vWF in cells cultured under normoxia or hypoxia in endothelial differentiation media (magnification, × 400). Scale bar: 5 *μ*m. (**f**) Tube formation in EPCs treated with or without ADSCs at a ratio of 1 : 1 and cultured in serum-free endothelial basal medium (EBM) under conditions of hypoxia (magnification, × 200). Scale bar: 20 *μ*m. (**g**) The length of formed tubes was quantified by densitometry. The EPC group treated for 1 day was used as a positive control (*n*=6). *P<0.05, **P<0.01, ***P<0.001. NS, no significant difference

Isolated ADSCs were labeled with different cell surface markers to determine cell phenotypes. ADSCs were positive for the mesenchymal stem cell (MSC) markers CD29, CD90, CD44 and CD105 and negative for the endothelial markers CD34 and vWF (Figure 4a). Figure 4b shows representative phase contrast images of ADSCs cultured under normoxic or hypoxic conditions for different times, which indicated that hypoxia promoted the growth of ADSCs. To determine the differentiation potential of ADSCs under different conditions, ADSCs were cultured under adipogenic, endothelial or osteogenic induction conditions and stained with Oil-red O and ALP. The results showed that hypoxia promoted the adipogenic and osteogenic differentiation potential of ADSCs at 1 and 3 weeks of culture (Figures 4c and d). Quantitative analysis was performed by measuring the absorbance of isolated fatty drops and by ALP activity assay, which confirmed that hypoxia increased the adipogenic and osteoblastic differentiation of ADSCs (Figures 4c and d). The endothelial differentiation of ADSCs was examined by immunofluorescence analysis of cells stained against vWf, which showed that hypoxia accelerated the differentiation of ADSCs into endothelial cells at 1 week (Figure 4e). ADSCs also induced tube formation from EPCs under hypoxic conditions and delayed the regression of capillary tubes on day 5, as determined using an *in vitro* angiogenesis assay and measurement of tube length (Figures 4f and g). Taken together, these results indicate that low oxygen tension increases the multipotent differentiation potential of ADSCs and suggest that ADSCs affect angiogenesis.

### Modulation of hypoxia-induced inflammatory cytokine, growth factor and ROS generation by Nrf2 and TLR4

**Figure 5:**
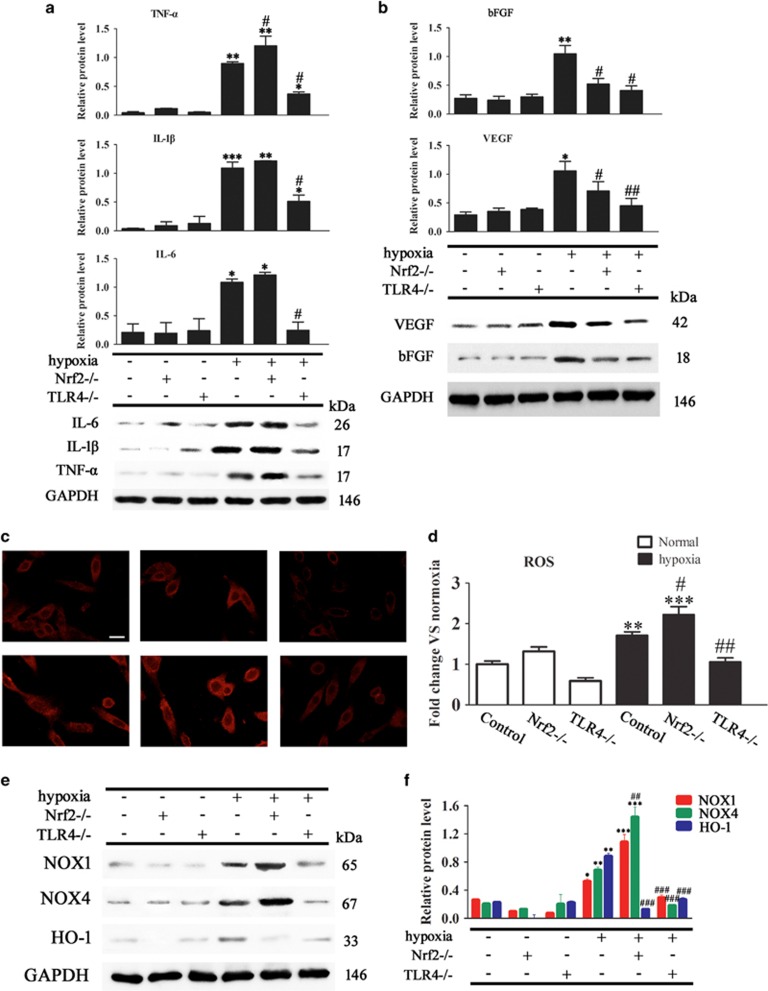
Effect of Nrf2 and TLR4 on the expression of inflammatory cytokines, growth factors, and intracellular and mitochondrial ROS generation in ADSCs. ADSCs were isolated from WT, Nrf2^−/−^ or TLR4^−/−^ mice and exposed or not to hypoxic conditions. (**a** and **b**) TNF-*α*, IL-1*β*, IL-6, VEGF and bFGF expression in ADSCs was measured by western blotting (*n*=5). (**c**) Micrographs of ADSCs labeled with dihydroethidium to detect ROS (magnification, × 40). Scale bar: 20 *μ*m. (**d**) Quantification of mitochondrial DCF fluorescence intensity in ADSCs (*n*=5). (**e**) NADPH oxidase (NOX) expression was measured by western blotting (*n*=5). (**f**) Relative protein expression and fold change of NOX1, NOX4 and HO-1 relative to GAPDH (*n*=3). *P<0.05, **P<0.01, ***P<0.001 *versus* the control group, and ^#^*P*<0.05, ^##^*P*<0.01, ^###^*P*<0.001 *versus* the hypoxia control group

We further examined the effect of Nrf2 and TLR4 on the release of inflammatory cytokines and growth factors from ADSCs isolated from WT, Nrf2^−/−^ or TLR4^−/−^ mice exposed or not to hypoxic conditions. Western blot analysis showed that the upregulation of TNF-*α*, IL-1*β* and IL-6 caused by hypoxia was enhanced by Nrf2 deletion, whereas it was significantly inhibited by TLR4 deletion (Figure 5a). Hypoxia induced the upregulation of bFGF and VEGF, and this effect was suppressed by the deletion of both Nrf2 and TLR4 (Figure 5b). Figure 5c shows representative images of DCF-DA-stained transplanted fat tissue sections for the detection of ROS. To quantify the changes in ROS levels in response to hypoxia, mitochondrial DCF fluorescence intensity was measured in ADSCs, which showed that hypoxia increased ROS generation, and this effect was enhanced by Nrf2 deletion and suppressed by TLR4 deletion (Figure 5d). Western blot analysis of ADSC lysates showed that TLR4 knockout suppressed, whereas Nrf2 knockout enhanced the hypoxia-induced upregulation of NOX1 and NOX4; however, both deletion of TLR4 and Nrf2 suppressed hypoxia-induced HOX-1 upregulation (Figures 5e and f). Taken together, these results suggested that the effects of hypoxia on the expression of inflammatory cytokines, growth factors and ROS generation were modulated by Nrf2 and TLR4.

### Nrf2 and TLR4 modulate ADSC apoptosis and ADSC-induced angiogenesis under conditions of hypoxia

**Figure 6:**
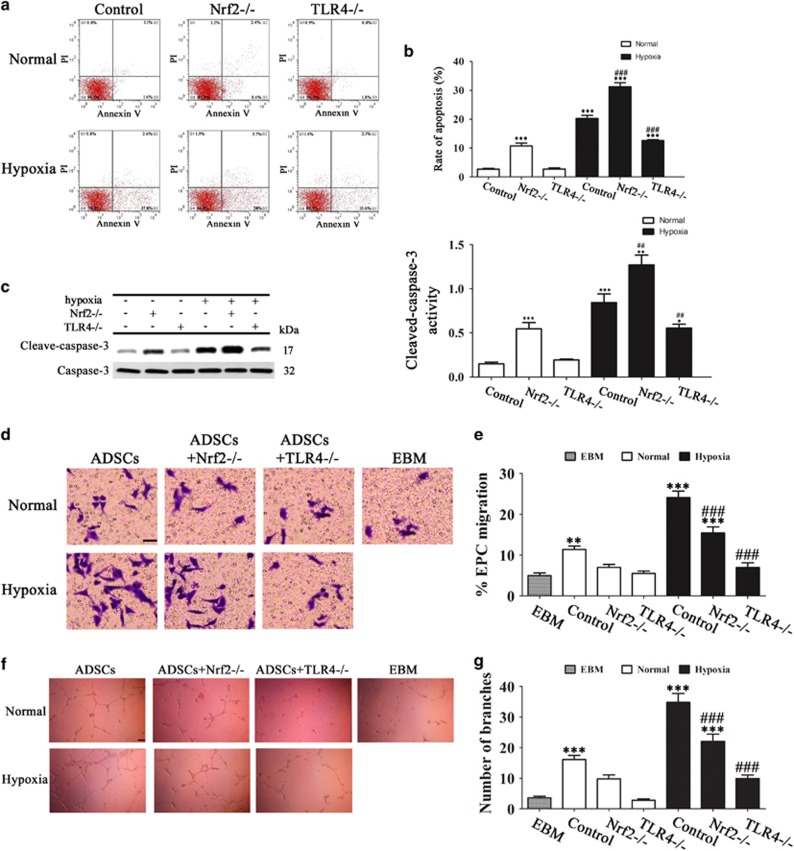
Nrf2 and TLR4 modulate ADSC apoptosis and ADSC-induced angiogenesis under conditions of hypoxia. ADSCs were isolated from WT, Nrf2^−/−^ or TLR4^−/−^ mice and cultured under normoxic or hypoxic conditions. (**a**) The effect of Nrf2 and TLR4 on hypoxia-induced apoptosis in ADSCs was analyzed by FITC-Annexin V and PI at 72 h after induction in hypoxia. In each panel, the Q4 (lower left) quadrant represents normal cells (untreated cells), the Q3 (lower right) quadrant contains early apoptotic cells (positive for AV and negative for PI), the Q2 (upper right) quadrant contains late apoptotic cells (positive for AV and positive for PI) and the Q1 (upper left) quadrant contains necrotic cells (negative for AV and positive for PI). (**b**) Quantification of the rate of Annexin V-positive cells at 72 h in hypoxia-induced ADSCs. Data represent the mean±S.E.M. from three replicate experiments determined by the Student's *t*-test. (**c**) Western blot detection of caspase-3 and cleaved caspase-3 in ADSCs cultured for 72 h in normoxic or hypoxic conditions; GAPDH was used as the loading control. Blot is a representative of three experiments. ***P<0.001 *versus* the control group, and ^##^*P*<0.01, ^###^*P*<0.001 *versus* the hypoxia control group. (**d**–**g**) The effect of Nrf2 and TLR4 deletion on EPC migration and angiogenesis induced by paracrine release from ADSCs cultured under hypoxia was evaluated. (**d**) Representative images of migrated EPCs in response to conditioned media (CM) from ADSCs or EBM (negative control). (magnification, × 200). Scale bar: 10 *μ*m. (**e**) Percentage of migrated EPCs toward CM. (**f**) CM from hypoxia-induced ADSCs led to the formation of interconnected tubular structures by EPCs (magnification, × 200). Scale bar: 20 *μ*m. (**g**) The number of branch points formed by EPCs in response to CM in all groups. **P<0.01, ***P<0.001 *versus* EBM; ^###^*P*<0.001 *versus* normal-ADSC-CM only

Detection of apoptosis by Annexin V/PI staining and flow cytometry and quantification of the rate of apoptosis showed that the increase in the rate of apoptosis induced by hypoxia was significantly enhanced by Nrf2 deletion and suppressed by TLR4 knockout (Figures 6a and b). Consistently, western blotting and densitometric quantification of bands showed that hypoxia increased the levels of cleaved caspase-3, indicating the induction of apoptosis, and this effect was significantly enhanced by Nrf2 deletion, whereas it was suppressed by TLR4 deletion (Figure 6c). Taken together, these results suggest that hypoxia-induced apoptosis in ADSCs is modulated by Nrf2 and TLR4. The chemotaxis of EPCs was assessed by measuring the migration of cells towards basal medium (endothelial basal medium (EBM)) or ADSC conditioned medium and tube formation, which are key steps involved in angiogenesis. The results showed that the increased migration of EPCs towards ADSC paracrine factors induced by hypoxia was suppressed significantly by deletion of Nrf2 and TLR4, with an approximately 50% inhibition of EPC migration in the TLR4^−/−^ group compared with the control (Figures 6d and e). Consistently, tube formation in EPCs was induced by ADSC conditioned medium under hypoxia, and this effect was significantly suppressed by Nrf2 or TLR4 knockout (Figures 6f and g). Taken together, these results indicate that the effect of ADSCs on promoting angiogenesis under hypoxia is modulated by Nrf2 and TLR4.

### Effect of hypoxia on the Keap-1/Nrf2/HO-1 and TLR4/NF*κ*B signaling pathways in ADSCs

**Figure 7:**
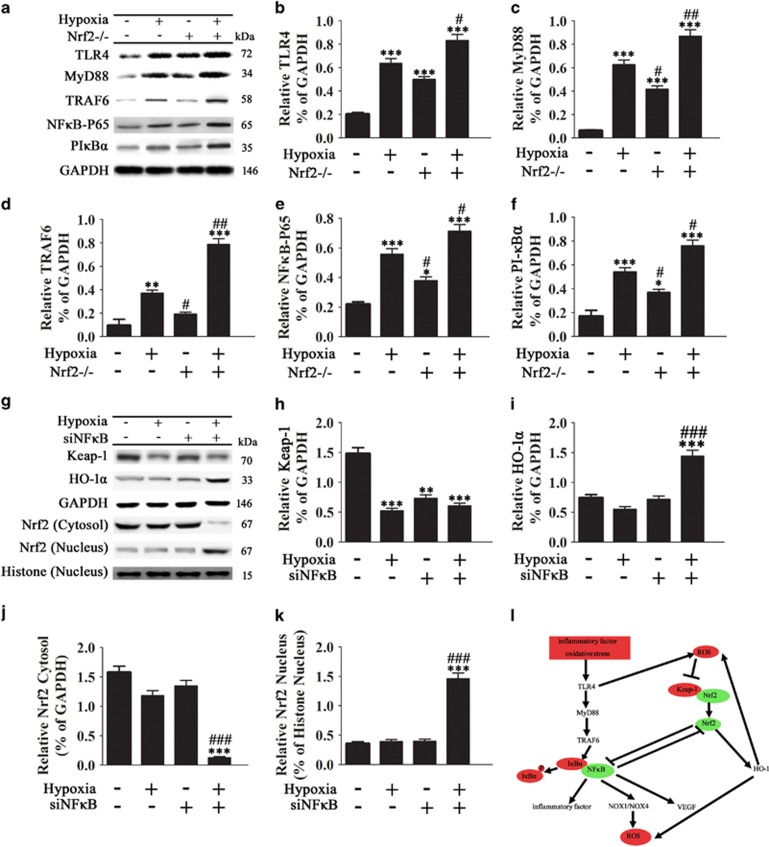
Effect of hypoxia on the Keap-1/Nrf2/HO-1 and TLR4/NF*κ*B signaling pathways in ADSCs. (**a**–**k**) ADSCs were cultured under hypoxic conditions for 48 h, and the protein expression of TLR4, MyD88, TRAF6, NF*κ*B p65, phosphorylated I*κ*B*α*, Keap-1 and Nrf2 was assessed by western blotting and quantified relative to GAPDH or histone expression (*n*=5, *P<0.05, **P<0.01, ***P<0.001 *versus* the control group, ^#^*P*<0.05, ^##^*P*<0.01, ^###^*P*<0.001 *versus* the hypoxia group. (**l**) Proposed schematic of the mechanisms regulating ADSC survival under conditions of hypoxia and the roles of TLR4 and Nrf2

TLRs act as sensors that detect microbial signals and elicit innate immune responses, and TLR signaling results in the activation of the transcription factor nuclear factor-*κ*B (NF-*κ*B) and the subsequent activation of inflammatory cytokine genes.^17^ We therefore examined the effect of Nrf2 and TLR4 on the TLR4/NF-*κ*B pathway under conditions of hypoxia by western blotting. The results showed that the upregulation of TLR4, MyD88 (a TLR4 adaptor), TRAF6 (a ring-domain E3 ubiquitin ligase that mediates the translocation of NF-*κ*B to the nucleus), the NF-*κ*B p65 subunit, and PI-*κ*B*α* induced by hypoxia was significantly enhanced by Nrf2 knockout (Figures 7a–f). Conversely, silencing of NF-*κ*B (Supplementary Figure S10) significantly upregulated HO-1 under conditions of hypoxia and maintained hypoxia-induced Keap-1 degradation (Figures 7h and i). NF-*κ*B knockdown significantly promoted the translocation of Nrf2 from the cytoplasm to the nucleus under conditions of hypoxia (Figures 7j and k). Taken together, these results indicated that hypoxia promoted Nrf2 translocation to the nucleus and the upregulation of HO-1 via the inhibition of NF-*κ*B nuclear translocation in ADSCs. Figure 7l shows a schematic describing the potential mechanism underlying the roles of Nrf2 and TLR4 on the enhanced survival of ADSCs under hypoxic conditions.

## Discussion

ADSCs are considered an important source of stem cells for tissue repair and regeneration and have been shown to increase the survival of fat grafts.^4^ Culture of ADSCs under hypoxic conditions increases their regenerative potential, and this effect was shown to be mediated in part by the stimulatory effect of ROS.^11, 18^ In addition, ADSCs are exposed to hypoxia and inflammation after transplantation, and understanding the effects of the inflammatory and hypoxic environment on ADSC viability is important to assess their effect on fat transplants. Here, we used TLR4^−/−^ and Nrf2^−/−^ mice to explore the mechanisms underlying the effect of ADSC enrichment on the survival of fat grafts *in vivo* and *in vitro*.

The results of the present study showed that ADSCs increased the survival of fat grafts after 2 months and this effect was attenuated in TLR4^−/−^ and Nrf2^−/−^ mice. The addition of ADSCs downregulated NOX1 and NOX4, and upregulated HO-1, and these effects were dependent on Nrf2 expression, indicating that the protective effect of Nrf2 against oxidative stress was mediated by the modulation of HO-1 and NOX1/4 expression. Nrf2 acts as a sensor of oxidative stress and therefore plays an important role in redox homeostasis and in the protection of cells against toxic insults and pathogen-associated diseases.^19^ However, Nrf2 activation is frequently present in cancer, and pharmacological targeting of the Keap-1–Nrf2 system has been investigated as a therapeutic strategy.^20^ Despite the known protective effect of Nrf2, few studies have assessed its potential to improve stem cell therapy. The effect of Nrf2 modulation was explored in a study in which Nrf2-overexpressing neuronal progenitor cells were transplanted into the striatum, which resulted in enhanced neuroprotective effects.^21^ Nrf2 plays a role in the regulation of cell fate determination in hematopoietic stem cells.^22^ Nrf2 activation by SIRT6 was shown to protect stem cells from oxidative stress-associated decay, revealing a mechanism by which SIRT6 forms a complex with Nrf2 and RNA polymerase II that is required for the expression of HO-1.^23^ Nrf2-overexpressing MSCs produce high levels of antioxidants, including SOD and HO-1, and show reduced apoptosis in response to oxidative stress, suggesting that Nrf2 overexpression could be a strategy for the prevention of graft cell death in MSC-based cell therapy.^24^ Our results support the modulation of the Keap-1–Nrf2 system as a strategy to improve the effect of ADSCs on fat grafts, as the effects of ADSCs on the viability and neovascularization of fat grafts, the production of NOX1/4 and HO-1, and ROS generation were reversed in the absence of Nrf2. Nrf2-activating compounds are currently being tested in clinical trials or are already in clinical use, such as dimethyl fumarate (Tecfidera), an Nrf2 pathway inhibitor used for the treatment of multiple sclerosis.^25^ Further studies are necessary to examine whether Nrf2 overexpression or activation of the Nrf2/Keap-1 system could improve the effect of ADSCs on the viability of fat grafts.

Assessment of fat transplants in our mouse model showed that ADSCs increased the vascularization of transplanted adipose tissue, upregulated VEGF and decreased ROS production, and these effects were dependent on TLR4 and Nrf2 expression. The increase in the number of capillaries induced by ADSC enrichment was partially suppressed by TLR4 and Nrf2 deletion. Nrf2 was previously shown to play an important role in the maintenance of the endothelial phenotype and the functional integrity of the vasculature.^26, 27^ Redox signaling pathways and ROS play a central role in angiogenesis and VEGF-mediated signal transduction.^28, 29^ VEGF increases ROS production in endothelial cells by activating NOX enzymes, and increased ROS activates Nrf2 by facilitating its dissociation from Keap-1, highlighting the role of Nrf2 in the regulation of angiogenesis via VEGF.^30, 31^ Nrf2 silencing impairs the angiogenic process in VEGF-stimulated human coronary arterial endothelial cells *in vitro*, impairing migration and the ability of cells to form capillary-like structures, demonstrating the essential role of Nrf2 in angiogenesis.^30^ Furthermore, Nrf2 is involved in angiogenic growth factor signaling by regulating the activity of the serine/threonine kinase domain of growth factor receptors or by modulating HIF-1*α*-dependent pathways, illustrating the role of hypoxia in the induction of Nrf2-dependent pathways.^32, 33^

The role of hypoxia in the regulation of ADSC differentiation and viability by Nrf2 and TLR4 was assessed in the present study by subjecting ADSCs to hypoxia/serum deprivation conditions to simulate ischemic conditions. Our results showed that hypoxia enhanced the proliferation of ADSCs and their differentiation in response to different induction media. ADSCs cultured under hypoxic conditions showed increased inflammatory cytokine and growth factor receptor expression and increased ROS production, and these effects were dependent on Nrf2 and TLR4 expression. Nrf2 knockout enhanced hypoxia-induced inflammatory cytokine expression, ROS production and apoptosis in ADSCs, whereas TLR4 knockout inhibited them, and both Nrf2 and TLR4 knockout suppressed the increase in growth factor levels induced by hypoxia. Furthermore, ADSCs promoted EPC migration and angiogenesis under conditions of hypoxia, and this effect was inhibited by Nrf2 and TLR4 deletion, indicating the involvement of the crosstalk between Nrf2 and TLR4 in hypoxia-induced EPC chemotaxis. TLR signaling can be affected by the cellular redox state, and Nrf2 plays an important role in ROS-mediated TLR4 activation and in modulating TLR4-driven inflammatory responses.^34, 35, 36^ Nrf2 and TLRs are linked in the regulation of innate immune responses, as Nrf2 maintains redox homeostasis during sepsis and restrains the dysregulation of proinflammatory signaling pathways.^37^ Both TLR and Nrf2 signaling are activated during inflammation, and TLRs trigger Nrf2 signaling in response to inflammation; however, little is known about the crosstalk between these innate immune components. A recent study showed that TLR signaling activates the Nrf2 pathway by promoting Keap-1 degradation through the autophagy pathway.^38^ TLR-7 activation was shown to result in Nrf2-mediated antioxidant protection against ROS through myeloid differentiation primary response gene 88, a TLR adaptor protein that activates NF-*κ*B.^39^

The effect of hypoxia on enhancing the viability and regenerative potential of ADSCs has been shown previously, and culture of ADSCs under hypoxic conditions increases their proliferation, migration and growth factor secretion.^11, 12, 40^ The stimulatory effect of hypoxia on ADSCs is partially mediated by the production of ROS,^41^ and ROS generated through the action of NOX enzymes are involved in redox signaling associated with stem cell mobilization and proliferation.^42^ In addition, VEGF, which functions in angiogenesis, is regulated by ROS and acts as a paracrine mediator of ADSC function.^41^ Our results suggest that modulation of Nrf2 and TLR4 signaling could be developed as a strategy to improve the angiogenic potential of ADSCs and thus increase their efficacy for use in regenerative medicine.

TLR4 signaling cascades lead to the activation of NF-*κ*B and the induction of proinflammatory cytokines and chemokines,^17^ and hypoxia plays an important role in the modulation of NF-*κ*B activity and the inflammatory response.^43^ Our results showed that hypoxia activated NF-*κ*B signaling and this effect was enhanced by Nrf2 deletion. Furthermore, silencing of NF-*κ*B promoted Nrf2 nuclear translocation and antioxidant responses under conditions of hypoxia. Taken together, these results suggest that crosstalk between Nrf2 and NF-*κ*B dependent signaling pathways modulates hypoxia-induced inflammation in ADSCs. These results support the potential of Nrf2 modulation to improve ADSC viability.

The present results led us to propose a model to explain the involvement of TLR4 and Nrf2 in the modulation of inflammation and oxidative stress related responses in ADSCs. In this model, inflammatory stimuli and oxidative stress stimulate TLR4 signaling and NF-*κ*B mediated expression of inflammatory cytokines, leading to the activation of NOX enzymes and the generation of ROS. TLR4 activation and ROS generation promote Nrf2 nuclear translocation and antioxidant responses while inhibiting NF-*κ*B signaling and inflammatory responses. Taken together, our results suggest a potential mechanism underlying the effects of inflammation and oxidative stress on ADSC viability and function via crosstalk between Nrf2, TLR4 and the NF-*κ*B signaling pathway. These results provide insight into the pathways regulating the responses of ADSCs to the transplantation microenvironment, which may be of value for the design of strategies based on the use of ADSCs in cell therapy.

## Materials and Methods

### Reagents

Antibodies against KDR, CD29, CD90, CD44, CD105, CD34, Von Willenbrand factor (vWF), GAPDH, NOX1, NOX4, HO-1, TLR4, MyD88, TRAF6, NF*κ*B P65, p-I*κ*B*α*, Keap-1, Nrf2 and 2′,7′-dichlorofluorescein diacetate (DCF-DA) were obtained from Sigma (St Louis, MO, USA). DMEM (high glucose), serum-free EBM, and fetal bovine serum (FBS) were from Nego (Shanghai, China). Cell lysis buffer (10 ×) was obtained from Cell Signaling Technology (CST, Boston, USA). The RT-PCR kit was purchased from TOYOBO (Shanghai, China). Other reagents included DAPI (Roche, Basel, Germany), hematoxylin and eosin (H&E; Toronto Chemicals, Toronto, ON, Canada), and trypsin (Sigma). All pairs of real-time PCR primers were synthesized by Shenggong Biotechnology (Shanghai, China). Other chemicals and reagents were of analytical grade.

### Animals and ethical statement

WT and Nrf2^−/−^ C57BL/6 mice were kindly provided by Dr. Masayuki Yamamoto from Tohoku University of Riken Resource Center. TLR^4lps–del^ (TLR4^−/−^) C57BL/10 mice were purchased from the Model Animal Research Center of Nanjing University (MARC). Six-week-old WT, TLR4^−/−^ and Nrf2^−/−^ mice were used in this study. All animals were treated in accordance with the Guide for the Care and Use of Laboratory Animals, and all experiments were approved and performed according to the guidelines of the Ethics Committee of Union Hospital of Fujian Medical University, Fuzhou, Fujian, China.

### Isolation, culture and identification of ADSCs

AD-MSCs were isolated as previously described.^44^ In brief, adipose tissue was harvested from normal, TLR4^−/−^ and Nrf2^−/−^ mice. Then, the tissue was washed with phosphate-buffered saline (PBS) and mechanically chopped before digestion with 0.2% collagenase I (Sigma) for 1 h at 37 °C with intermittent shaking. The digested tissue was washed with Dulbecco's modified Eagle's medium (DMEM) (Sigma) containing 15% FBS, and then centrifuged at 1000 r.p.m. for 10 min to remove mature adipocytes. The cell pellet was resuspended in DMEM supplemented with 15% FBS, 100 U/ml penicillin and 100 *μ*g/ml streptomycin in a 37 °C incubator with 5% CO_2_. ADSCs reaching 80–90% confluency were detached with 0.02% ethylenediaminetetaacetic acid/0.25% trypsin (Sigma-Aldrich) for 5 min at room temperature and then re-plated. For phenotypic analysis, fluorescein isothiocyanate (FITC-F) or phycoerythrin was used. The expression of the following markers was investigated: CD29, CD90, CD44, CD105, CD34 and vWF. An IgG-matched isotype served as the internal control for each antibody. For normoxic cultures, ASCs were cultured in 95% air (20% O_2_) and 5% CO_2_. For hypoxia studies, ASCs were cultured in a multigas incubator (ASTEC) that was flushed with a humidified gas mixture composed of 2% O_2_:5% CO_2_:93% N_2_.

### Growth potential

To test the effect of hypoxia on the growth of ADSCs, ADSCs were cultured in a multigas incubator (ASTEC) that was flushed with a humidified gas mixture composed of 2% O_2_:5% CO_2_:93% N_2_. For normoxic cultures, ADSCs were cultured at 37 °C and 5% CO_2_. All experiments were performed at least three times. To assess growth potential, ADSCs were thawed, counted and plated onto a 100-mm dish at a density of 1 × 10^5^ cells/dish. Three dishes were placed under normoxic conditions and another three in each group (contain normal, Nrf2^−/−^ and TLR4^−/−^ groups) under hypoxic conditions. The cells were allowed to proliferate for 5 days (P1–P8) and samples were harvested with 0.05% trypsinization. Cell numbers of ADSCs were determined using a hemocytometer. Cells were resuspended in 10 ml of control medium, and then re-plated at a density of 1 × 10^5^ cells/dish; the procedure was repeated with hypoxic cells under hypoxia and with normoxic cells under normoxia from day 0 to 40, and cell numbers were determined at harvest for each 5 days.

### ELISA for soluble inflammatory cytokines and growth factors

The amounts of IL-6, IL-1*β*, TNF-a, VEGF and bFGF in the supernatants of ADSCs or serum from mice with or without a fat graft were measured using commercially available ELISA kits (Sen-Xiong Company, Shenzhen, China). In accordance with the manufacturer's instructions, supernatants were stored at −80 °C before measurement and both standards and samples were run in triplicate. OD450 was calculated by subtracting the background, and standard curves were plotted.

### Fat graft implantation

Inguinal fat pads were harvested and tissues were processed as described above. Adipose tissues (0.5 ml) with or without the addition of ADSCs (0.3 ml adipose tissue+0.2 ml ADSCs/GFP-ADSCs at 5 × 10^6^ cells/ml) were used for fat grafts in the corresponding genotype mice. Mice were aged 6 weeks regardless of gender. All surgical tools were sterilized prior to surgery. A No. 16 syringe needle (Shifeng, Chengdu, China) was used to inject 0.5 ml of fat particles subcutaneously into two recipient sites on the back of experimental mice. After the operation, mice were cared for at the animal center (SPF level) of Union Hospital of Fujian Medical University, Fuzhou, Fujian, China.

### Measurement of transplanted fat tissue ROS generation

For fat tissues, a DHE fluorescent probe was used for detecting ROS generation. Frozen sections were fixed with iced acetone at 4°C for 1 h. The sections were washed with PBS and incubated with the 10 *μ*M DHE fluorescent probe for 1 h at 37 °C. Tissue sections were visualized with a fluorescence microscope (ECLIPSE E600; Nikon, Tokyo, Japan). For cells, after the different pre-treatments, DCF-DA (10 *μ*M) was added to the medium for 30 min; cells were incubated at 37 °C in the dark, and then washed immediately with PBS. Fluorescent images of the cells were examined for the detection of ROS production with a fluorescence microscope (ECLIPSE E600; Nikon). The fluorescence intensity of DCF-DA was measured and calculated using flow cytometry (Flowjo, Ashland, OR, USA).

### Differentiation assays

ADSCs between 1 and 20 days were plated at a density of 3 × 10^4^ cells/cm^2^ on culture dishes in conditioned medium (CM). After 24 h, the medium was removed and cells were rinsed twice with DPBS. Adipogenic and endothelial differentiation were induced by culturing ADSCs for 1 or 3 weeks in Adipogenic Differentiation Media, Endothelial Differentiation Media and Osteogenic Differentiation Media (Cellular Engineering Technologies Inc., Coralville, IA, USA). Oil-red O and the Lipid Assay kit (Primary Cell Co., Ltd, Hangzhou, China) were used to analyze the differentiation of ADSCs into adipocytes. Quantification of fat drops was performed using the Lipid Assay kit. Endothelial cell differentiation was determined by vWF immunofluorescence staining. The Alkaline Phosphatase (ALP) Assay Kit (Primary Cell Co.) was used according to the manufacturer's instructions.

### Apoptosis assay

Apoptosis of ADSCs was measured by flow cytometry. ADSCs were harvested and resuspended in 500 *μ*l of binding buffer. Cells were then incubated with 5 *μ*l annexin V-FITC and 5 *μ*l propidium iodide (BD Biosciences, San Jose, CA, USA) at room temperature in the dark for 15 min. Subsequently, the samples were analyzed by flow cytometry using a FACSCalibur flow cytometer and FlowJo software (Flowjo).

### Chemotaxis effect assays

Cell chemotaxis assays were performed using Transwell chambers as described previously.^45^ Briefly, ADSCs and EPCs were seeded at a density of 2 × 10^5^ cells/well in six-well plates. After 48 h of hypoxia or normal induction of ADSCs, the CM was collected and added to the lower chamber. At the same time, after 48 h of normal induction, EPCs were trypsinized and resuspended in EBM. For the migration assay, a total of 1 × 10^5^ cells were added to the top chamber of prewetted Transwell inserts (BD Falcon 8 *μ*m control insert). After incubation for 16 h (migration assay), cells were washed, fixed with 10% formaldehyde for 20 min and stained with 0.5% crystal violet. Cells that did not migrate through the pores were mechanically removed using a cotton swab. The images of migrated cells were acquired using an inverted microscope with a magnification of × 200. The number of migrated cells was counted from five or six randomly selected fields in a blind manner. All migration experiments were performed in triplicate and repeated three times.

### Evaluation of the angiogenic potential of ADSCs and the effect on EPCs under hypoxic conditions

To assess the effect of ADSCs on EPC tube formation under conditions of hypoxia, 1 × 10^4^ EPCs and 1 × 10^4^ ADSCs were mixed and resuspended in serum-free EBM, then seeded on Matrigel (around 50 *μ*l of Matrigel) in cold wells (maintained at 4 °C) in a 96-multiwell plate. After Matrigel jellification at 37 °C for 30 min, cells were seeded at a concentration of 2 × 10^4^ cells/well in 50 *μ*l. On days 1, 2 and 5 after seeding, the number of tubes formed was counted at × 10 magnification by inverted microscopy.

The effect of paracrine release from ADSCs (including WT, Nrf2^−/−^ and TLR4^−/−^) on EPC angiogenesis was examined on BD Matrigel wells (BD Biosciences). Prior to EPC seeding, 96-well microtiter plates were coated with 80 ml of Matrigel per well and incubated for 1 h. EPCs were suspended in CM from ADSCs or serum-free EBM and plated in triplicate at 12 000 cells per well. The formation of tube-like structures was examined at each hour for up to 12 h. Representative images were acquired and analyzed utilizing ImageJ software (NIH). Tube formation was analyzed by quantifying the number of branches or tube length per high-powered field.

### Histological analysis

At 2 months after fat graft, adipose tissues were excised, washed with saline solution and placed in 10% formalin, then cut into 4- to 5-*μ*m-thick sections and stained with H&E for histopathology, followed by visualization by light microscopy.

### RNA isolation and semiquantitative RT-PCR

Total RNA was isolated from various pretreated ADSCs using TRIzol according to the manufacturer's instructions (Invitrogen, Carlsbad, CA, USA). Equal amounts of RNA were added to a reverse transcriptase reaction mix (Thermo Scientific, MA, USA), with oligo-dT as a primer. The resulting templates were subjected to PCR using the following specific primers: GAPDH (sense 5′-CATCTTCTCAAAATTCGAGTGACAA-3′, antisense 5′-AGTAGACTCCACGACATACTCA-3′); NOX1 (sense 5′-ACCACTGGCTCTCAGTTTTG-3′, antisense 5′-TCGACACACAGGAATCAGGA-3′); NOX4 (sense 5′-GTACAACCAAGGGCCAGAATA-3′, antisense 5′-TCTTGCTTTTATCCAACAAT-3′); and HO-1 (sense 5′-CCAAGGAGGTACACATCCAA-3′, antisense 5′-GAGTGGGGCATAGACTGGGT-3′).

### Construction and infection

For knockdown of NF-*κ*B, small interfering RNA (siRNA) molecules (siNF-*κ*B) were synthesized by GenScript. The target sequence was 5′-GGACCTATGAGACCTTCAATT-3′. ADSCs were transfected with siRNA using the oligofectamine protocol according to the manufacturer's instructions (Invitrogen). A GFP-labeled vector was used to infect ADSCs to monitor the differentiation of ADSCs by immunofluorescence microscopy using a Fluoview 1000 System (Olympus, Irving, TX, USA).

### Western blot analysis

The protein samples extracted from ADSCs or adipose tissues were subjected to sodium dodecyl sulfate-polyacrylamide gel electrophoresis and transferred to a polyvinylidene difluoride membrane. Membranes were blocked with TBST containing 5% milk and incubated with the indicated primary antibodies overnight at 4 °C. The membranes were then incubated with horseradish peroxidase conjugated secondary antibodies and visualized using the enhanced chemiluminescence system. Densitometric analysis was performed using Scion Image software (Scion, Frederick, MD, USA).

### Immunofluorescence

ADSCs or adipose tissue slices were fixed with 4% paraformaldehyde for 15 min, permeabilized with 0.1% Triton X-100 for 10 min and blocked with 3% bovine serum albumin in PBS for 30 min. Cells were incubated with the indicated antibodies overnight followed by conjugated secondary antibodies for 1 h at room temperature in the dark. After several washes with PBS, the slides were incubated with DAPI for 3 min and then mounted in glycerol. Fluorescence was imaged with a fluorescence microscope.

### Statistical analysis

Results are expressed as mean±standard deviation (S.D.). Statistical significance was evaluated by analysis of variance followed by Tukey–Kramer multiple comparison test and by Student's *t*-test. A *P-*value of <0.05 denotes statistical significance.

## Abbreviations

ADSCs: autologous fat grafting
TLR4: Nrf2; inflammatory; oxidative
