# Supplementary material for: Adipose-derived mesenchymal stem cells promote the survival of fat grafts via crosstalk between the Nrf2 and TLR4 pathways
Source: Cell Death Dis. 2016 Sep 8;7(9):e2369–. doi: 10.1038/cddis.2016.261 (PMC5059864; doi:10.1038/cddis.2016.261)
Supplement: Supplementary Materials [file cddis2016261x1.doc]

**Materials and methods**

**Isolation and identification of EPCs**

Mononuclear cells were isolated from mouse umbilical cord blood. EPCs (CD133+) were selected using CD133-coupled magnetic microbeads (Miltenyi Biotech, Bergisch Gladbach, Germany), following the manufacturer’s instructions. After isolation, CD133+ cells were expanded in DMEM medium. Immunophenotypic analysis was performed by staining 5 × 10^5^ isolated and expanded EPCs. The cells were incubated with conjugated monoclonal antibodies against CD31, CD34 and KDR (Sigma, St. Louis, MO). Isotype-identical antibodies served as controls (PharMingen). For analysis of CD31, CD34 and KDR, the cells were further incubated with a biotinylated anti-mouse IgG (H1L) antibody made in horse (Vector Laboratories) and with FITC-conjugated streptavidin (Caltag, South San Francisco, CA). After treatment, the cells were fixed in 1% paraformaldehyde. Quantitative analyses were performed using a FACSCalibur flow cytometer and FlowJo software (Flowjo, Ashland, OR, USA). Logarithmic fluorescence intensity was recorded for 10,000–20,000 cells per sample.

**Green Fluorescent Protein (GFP) label ADSCs**

The isolated ADSCs were seeded into 24-well plates (5×10^4^ cells/well) with 10% FBS at 37ºC in an atmosphere containing 5% CO2. When ADSCs reached 70% confluence, lentivirus with GFP plasmid (Invitrogen) was added to the wells based on a multiplicity of infection (MOI) of 20. The medium was replaced at 24 h after infection and the fluorescence intensity was measured after 96 h. Non-infected ADSCs were used as negative controls.

**Supplementary figure legends**

Figure 8 Characteristics of EPCs. Flow cytometry histograms of EPCs for hematopoietic and stromal markers. Murine EPCs were grown under normoxic conditions in Dulbecco’s modified Eagle’s medium (DMEM)/10% fetal bovine serum. Primary EPCs expressed KDR and CD34 and did not express CD31. All experiments were performed in triplicate.

Figure 9 Fluorescence microscopy images of GFP-ADSCs. Bar, 100 μm. GFP: green fluorescent protein.

Figure 10 The relative protein expression and mRNA levels of NF-κB were measured in cells treated with siRNA against NF-κB by western blotting and real-time PCR, respectively. *p < 0.05 versus control.
