# Supplementary figures and images for: Adipose-derived mesenchymal stem cells promote the survival of fat grafts via crosstalk between the Nrf2 and TLR4 pathways
Source: Cell Death Dis. 2016 Sep 8;7(9):e2369–. doi: 10.1038/cddis.2016.261 (PMC5059864; doi:10.1038/cddis.2016.261)

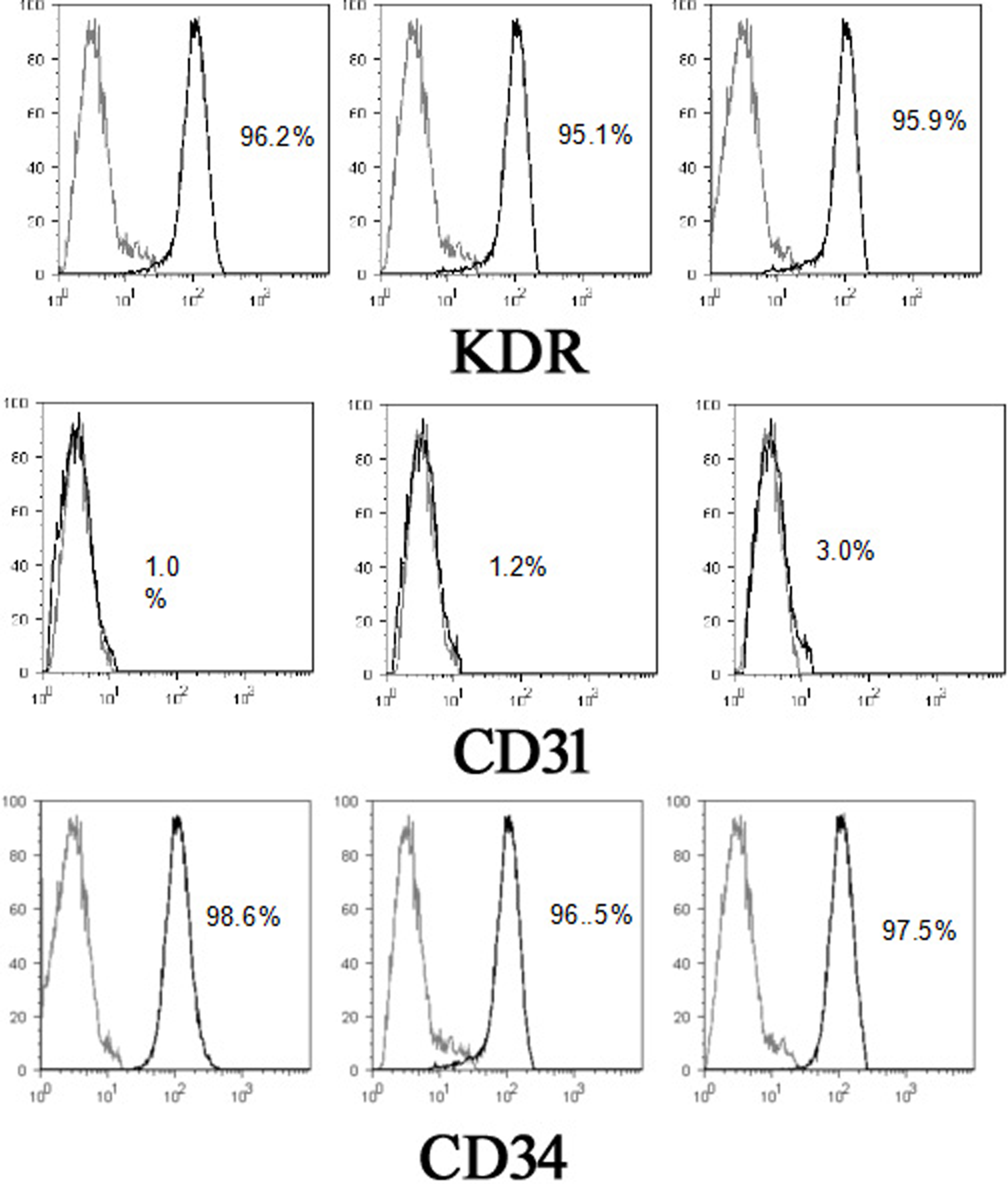

Supplement: Supplementary Figure 8 [file cddis2016261x2.tif]

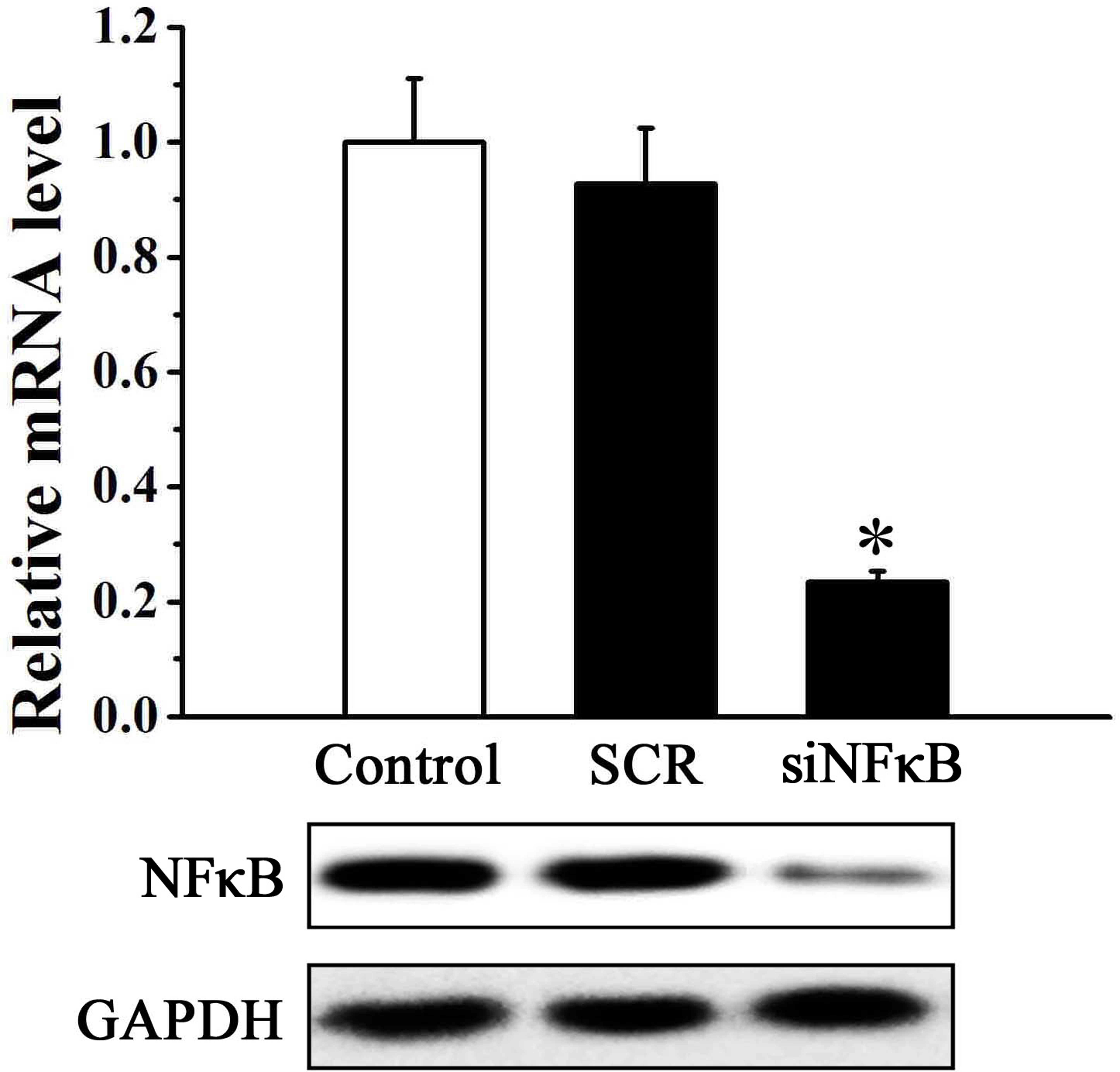

Supplement: Supplementary Figure 10 [file cddis2016261x4.tif]
